# Nano-silicone and *Ascophyllum nodosum*-based biostimulant down-regulates the negative effect of in vitro induced-salinity in *Rosa damascena*

**DOI:** 10.1186/s12870-023-04584-2

**Published:** 2023-11-14

**Authors:** Hanifeh Seyed Hajizadeh, Sahar Azizi, Ahmad Aghaee, Sinem Karakus, Ozkan Kaya

**Affiliations:** 1 Department of Horticulture, Faculty of Agriculture, University of Maragheh, Maragheh, 55136-553 Iran; 2 Department of Biology, Faculty of Science, University of Maragheh, Maragheh, Iran; 3 Çölemerik Vocational School, Hakkari University, Hakkari, 30000 Turkey; 4 Republic of Turkey Ministry of Agriculture and Forestry, Erzincan Horticultural Research Institute, Erzincan, 24060 Turkey; 5 Department of Plant Sciences, North Dakota State University, Fargo, ND 58102 USA

**Keywords:** Biostimulant, Nano particle, Osmolytes, Salt stress, Scavengers

## Abstract

**Background:**

*Rosa damascena* is extensively cultivated in various regions of Iran due to its aesthetic attributes, medicinal qualities, and essential oil production. This study investigated the efficacy of *Ascophyllum nodosum* extract (AnE) at concentrations of 0, 2, and 3 g L^− 1^ and Nano-silicon (nSiO_2_) at concentrations of 0, 50, and 100 mg L^− 1^ in ameliorating the impact of salinity on two genotypes of Damask rose (‘Chaharfasl’ and ‘Kashan’) under in vitro culture conditions. Additionally, various physio-chemical characteristics of *R. damascena* explants were assessed.

**Results:**

The findings revealed that exposure to 100 mM NaCl resulted in a substantial reduction in the Relative Water Content (RWC), Membrane Stability Index (MSI), leaf pigments (Chlorophyll *b*, Chlorophyll *a*, total Chlorophyll, and carotenoids), chlorophyll fluorescence parameters, and protein content in both genotypes when compared to control conditions. Salinity induced a significant increase in the parameter *F0* and a decrease in the parameter *Fv/Fm* compared to the control conditions in both genotypes. Nonetheless, the genotype Kashan treated with 3 g L^− 1^ AnE + 100 mg L^− 1^ nSiO_2_ exhibited the maximum *Fm* value under control conditions, with a significant difference compared to other treatments. Furthermore, salinity caused a considerable reduction in *Fm* in both ‘Kashan’ and ‘Chaharfasl’ by 22% and 17%, respectively, when compared to the control condition. ‘Kashan’ displayed the maximum *Fv/Fm* compared to the other genotype. The maximum levels of Malondialdehyde (MAD) and hydrogen peroxide (H_2_O_2_) were also observed in explants affected by salinity. The combination of 3 g L^− 1^ AnE + 100 mg L^− 1^ nSiO_2_, followed by 2 g L^− 1^ AnE + 100 mg L^− 1^ nSiO_2_, exhibited substantial positive effects. Salinity also led to an increase in proline content and the activity of peroxidase (POD), superoxide dismutase (SOD), guaiacol peroxidase (GPX), and catalase (CAT) in both genotypes. The activity of these enzymes was further enhanced when AnE was applied at concentrations of 2 and 3 g L^− 1^ in combination with 100 mg L^− 1^ nSiO_2_.

**Conclusions:**

The ‘Kashan’ genotype displayed greater tolerance to salinity by enhancing water balance, maintaining membrane integrity, and augmenting the activity of antioxidant enzymes compared to ‘Chaharfasl’. The utilization of nSiO_2_ and AnE biostimulants demonstrated potential benefits for *R. damascena*, both under salinity and control conditions. These findings hold substantial importance for researchers, policymakers, and farmers, offering valuable insights into the development of salinity-tolerant crop varieties.

**Supplementary Information:**

The online version contains supplementary material available at 10.1186/s12870-023-04584-2.

## Background

*Rosa damascena*, recognized as the national flower of Iran, is primarily cultivated to produce flowers, specifically for the extraction of rose water (Golab) and essential oil [1]. These flowers’ hips are rich in essential nutrients such as ascorbic acid, carotene, and various minerals, including iron, calcium, manganese, potassium, sodium, phosphorus, and zinc [2]. However, the detrimental impact of salinity on agricultural crops, particularly in semi-arid and arid regions, has emerged as a significant environmental factor affecting the cultivation of *R. damascena* [3]. Salinity affects more than 900 million hectares of land worldwide, encompassing 20% of all agricultural land [4]. Research by Ali et al. [5] demonstrated that salinity leads to a considerable decrease in some factors including leaf area index, plant height, and the number of adventitious shoots in *R. damascena*. Furthermore, salinity impedes the reproductive phase, resulting in a substantial reduction in flower yield, leaf relative water content (RWC), and the dry weight of *Rosa chinensis* [6]. The accumulation of salts induces ion toxicity, disrupts nutrient balance, creates osmotic stress, and leads to water deficiency [3]. Consequently, the limited growth of plants under salinity can’t be attributed to a single physiological process. Metabolic description for the mentioned process encompass a reduction in water potential, stomatal closure, diminished photosynthetic activity, impaired nutrient absorption, inhibited growth of leaves and fruit, and alterations in enzyme and gene signaling [7]. Previous studies have established a salt tolerance threshold for *Rosa* is approximately 7–8 dS m^− 1^ [8, 9], while *R. damascena* ‘Kashan’ exhibiting a threshold of about 6.5 dS m^− 1^ [10].

The tolerance of plants to salinity is contingent upon various factors, including the plant species, prevailing weather conditions, irrigation methods, and the type of soil or substrate [11]. Consequently, there is a pressing need for research aimed at not only developing strategies to replace conventional chemical agents with natural or biological alternatives, such as plant bio stimulants but also reducing the reliance on chemical inputs. Plant bio stimulants have garnered significant attention in modern agriculture as a means to increase crop productivity, bolster resistance to exogenous stresses, and optimize nutrients utilization [12]. Indeed, the recent advances in innovative technologies centered around biological resources, including biostimulants, represent an effective avenue for improving crop performance, and these methods have been empirically validated [13]. Biostimulants derived from organic sources, often employed in limited quantities, exhibit remarkable potential for promoting the growth and development of various crops, both under desired conditions and in the face of stressful environmental factors. Among these substances, silicon has been recognized for its capacity to mitigate the impact of many biotic and abiotic stressors [14]. Silicon’s beneficial effects may stem from its ability to reduce transpiration under abiotic stress conditions and to partially obstruct vessels post-transpiration [15]. Researchers have taken particular interest in evaluating the effects of silicon, especially given that many ornamental plants are cultivated in soilless systems with minimal silicon concentrations [16]. The application of silicon to ornamental plants has demonstrably improved their quality, resilience, and overall flowering performance [17]. In contrast to chemical alternatives, seaweed extracts not only prevent environmental degradation but are also non-toxic, posing no threat of hazardous pollution to humans, animals, or birds [18]. The bioactive compounds derived from seaweed are widely employed in agricultural and horticultural products, resulting in increased yields and improved product quality [19]. Some plants, such as algae, naturally produce growth-promoting compounds, leading to the utilization of their extracts in the production of fertilizers that enhance plant growth and yield. Notably, *Ascophyllum nodosum*, a type of algae, has been found to increase chlorophyll levels in plant leaves and boost the presence of amylase enzymes in plant organs, facilitating the breakdown of unusable glucosides within the plant [20]. The application of algae extracts promotes numerous positive effects, such as increased plant growth, leaf numbers, root stimulation, accelerated flowering, enhanced fruit production, delayed leaf aging, improved stress tolerance (including salinity, drought, and temperature), and elevated fruit quantity and quality [21]. Furthermore, these algae extracts are known to be rich in potassium [22].

Biostimulants have the potential to cope the adverse effect of abiotic stresses through the activation of stress-related genes and antioxidative compounds [23]. Silicon, in particular, possesses the capability to directly and indirectly stimulate plant growth and performance under a various abiotic and biotic stress conditions [15, 24]. Satisfactory outcomes from in vitro culture with silicon supplementation have been observed in plants such as begonia, orchid, and anthurium [25–27]. Hence, the in vitro culture method is considered an advanced tool for rapidly and precisely assessing plant responses to experiments. Given our prior research demonstrating the positive effects of nSiO_2_ under water deficiency [28], the primary aim of the present experiment was to compare the efficacy of *Ascophyllum nodosum* extract (AnE) and nano-silicon (nSiO_2_) in influencing certain physico-chemical characteristics of *R. damascena* under in vitro-induced salinity. This study also aimed to elucidate the mechanisms by which bio stimulants modulate the impact of salt stress.

## Results and discussion

### Physiological traits

**Fig. 1:**
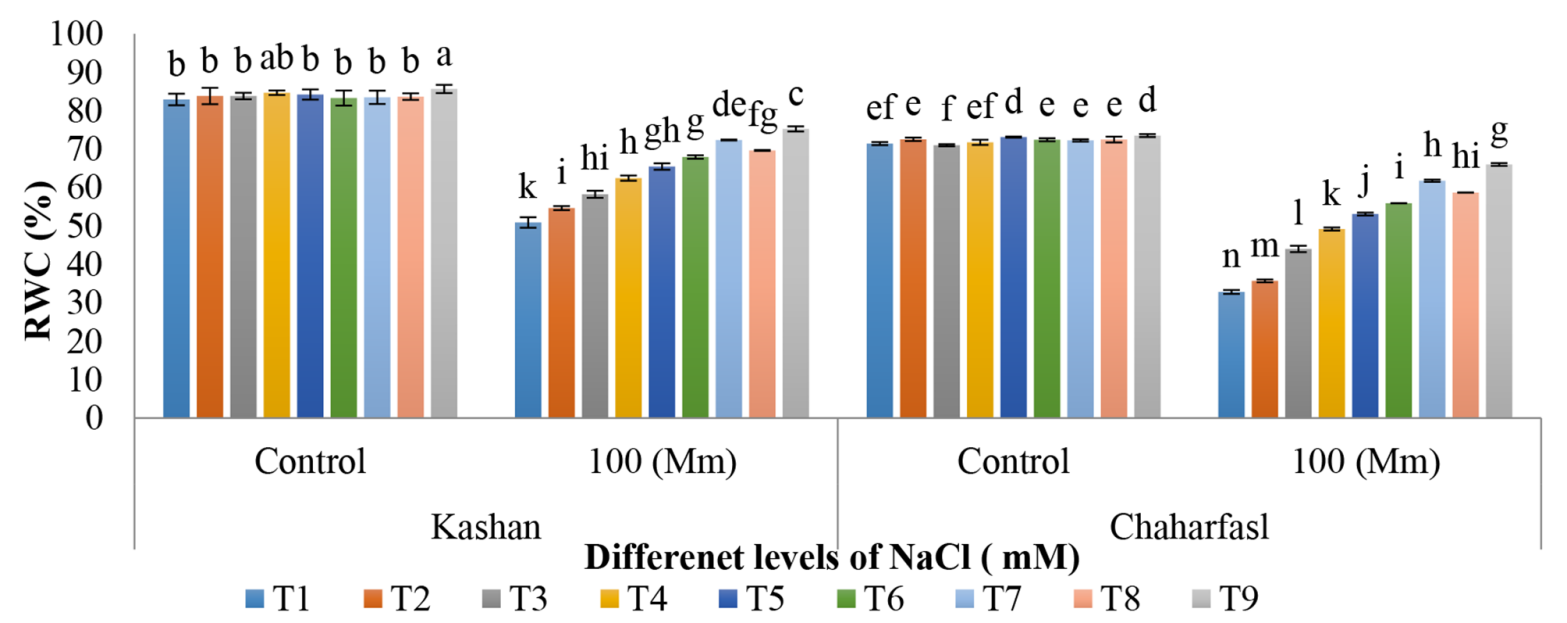
The interaction of in vitro induced-salt stress, nSiO_2_, and AnE on leaf RWC in two genotypes of *R. damascena*; **T1** (Control), **T2** (AnE 2 g/L), **T3** (AnE 3 g/L), **T4** (nSiO_2_ 50 mg/L), **T5** (nSiO_2_ 100 mg/L), **T6** (AnE 2 g/L + nSiO_2_ 50 mg/L), **T7** (AnE 2 g/L + nSiO_2_ 100 mg/L), **T8** (AnE 3 g/L + nSiO_2_ 50 mg/L), **T9** (AnE 3 g/L + nSiO_2_100 mg/L)

Salt stress led to a decrease in RWC by 38% and 54% in ʻKashanʼ and ʻChaharfaslʼ, respectively compared to the control. Results of treatment with nSiO_2_ and AnE under salt stress on leaf RWC in two genotypes of Kashan and Chaharfasl (Fig. 1), showed that the maximum amount of RWC with a significant difference compared to other treatments was obtained in T4 (50 mg L^− 1^ nSiO_2_) and T9 (AnE 3 g L^− 1^ + nSiO_2_ 100 mg L^− 1^) in control explants of ‘Kashanʼ while ʻChaharfaslʼ had the least RWC under 100 mM salt stress. The most suitable treatment in both genotypes was concluded in combined application of AnE (3 g L^− 1^) and nSiO_2_ (100 mg L^− 1^) as increased the RWC by 1.4 and 2 folded in ʻKashanʼ and ʻChaharfaslʼ, respectively. In addition, ʻKashanʼ was more tolerant to salt stress (at 100 mM NaCl) than ʻChaharfaslʼ because the RWC reduction rate in ʻKashanʼ was lower than the other ones.

RWC represents a pivotal factor in plant growth and development, particularly in the context of drought and salt stress. A higher RWC value is indicative of cell swelling and enlargement, thereby contributing to the progress of plant growth under such adverse conditions [29]. The intimate association between water uptake and RWC in leaves becomes evident when drought or salt stress occurs, resulting in a reduction in water uptake, which consequently leads to a decrease in RWC. RWC is closely intertwined with key physiological factors such as transpiration rate, stomatal conductance, and photosynthesis. As RWC diminishes, stomatal conductance and carbon dioxide diffusion are compromised, ultimately leading to a reduction in photosynthesis. Alterations in RWC content serve as a short-term response to stress and serve as a gauge of a plant’s ability to withstand stress conditions [30]. Silicon is postulated to elevate RWC in plants by curtailing transpiration and enhancing the water status of the plant, potentially resulting in an upsurge in photosynthesis. This effect is achieved through the deposition of silicon on the cell wall, particularly on epidermal cells, which mitigates water loss from these regions and impedes the movement of water along non-vascular pathways [31]. By depositing on the cell wall, especially epidermal cells, silicon reduces the loss of water from these parts and prevent of the movement of water from the non-vascular path [32]. Furthermore, seaweed extract has been noted to stimulate plant growth, foster root growth, augment water absorption, and elevate leaf RWC [33].

**Fig. 2:**
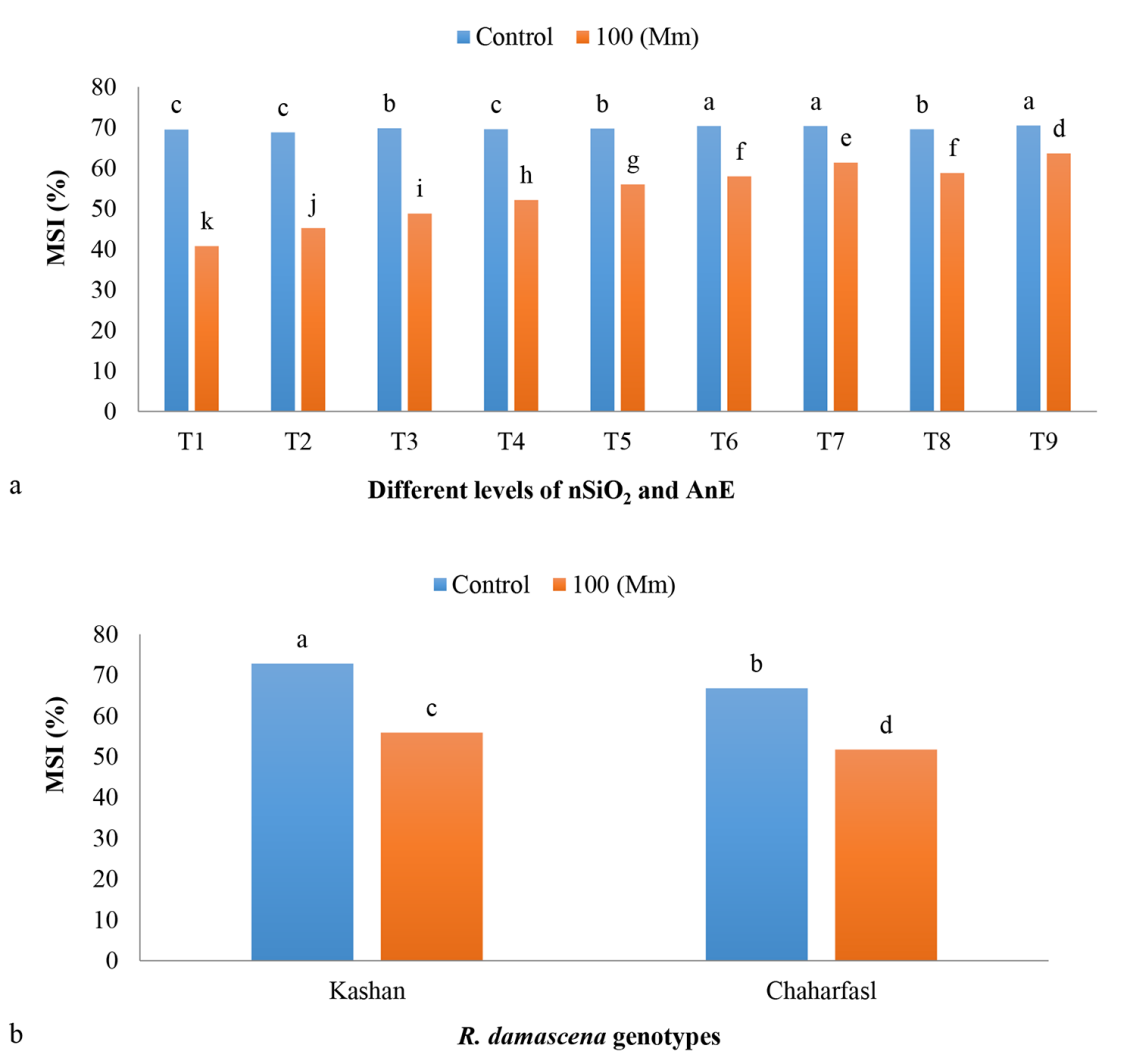
The interaction of in vitro induced-salt stress and **a)** SiO_2_-NPs, and AnE and **b)** *R. damascena* genotypes on MSI; **T1** (Control), **T2** (AnE 2 g/L), **T3** (AnE 3 g/L), **T4** (nSiO_2_ 50 mg/L), **T5** (nSiO_2_ 100 mg/L), **T6** (AnE 2 g/L + nSiO_2_ 50 mg/L), **T7** (AnE 2 g/L + nSiO_2_ 100 mg/L), **T8** (AnE 3 g/L + nSiO_2_ 50 mg/L), **T9** (AnE 3 g/L + nSiO_2_100 mg/L)

According to the Fig. 2a, the results showed that treatments T6 (AnE 2 g/L + nSiO_2_ 50 mg/L), T7 (AnE 2 g/L + nSiO_2_-NPs 100 mg/L), and T9 (AnE 3 g/L + nSiO_2_ 100 mg/L) had the maximum MSI value with a significant difference in comparison with the other treatments under control conditions (no salinity), while decreased significantly at 100 mM NaCl as the minimum value was related to the T1 (control). Results of the interaction between salt stress and *R. damascena* genotypes on MSI (Fig. 2b), showed that the maximum MSI value was observed in ʻKashanʼ under control conditions, while the least value was related to the ʻChaharfaslʼ at 100 mM NaCl. In general, ʻKashanʼ had the highest MSI and was more tolerant to salinity than the other genotype. Some researchers noted that silicon, as a messenger molecule, reduces the damage caused by free radicals of oxygen and ion leakage by increasing the plant’s antioxidant defense power [34]. In addition, part of the improvement of membrane’s stability due to the application of silicon can be related to the effect of this material in increasing the RWC [35]. It has been reported that seaweed extracts not only enhance leaf growth by facilitating the transport of osmolytes and ions that increase membrane permeability and water retention but also promote the accumulation of nonstructural carbohydrates, thus enhancing energy storage and metabolic processes [36].

Salt stress caused to reduce in the photosynthetic pigments including Chl *a*, Chl *b*, total Chl, and CARs, in comparison with the control conditions, since they are an indicator of stress. Photosynthesis for plants is the primary step for producing energy which is prevented by salinity because of the chlorophyll and carotenoids degradation by chlorophyllase activation [37]. Although salinity caused to reduce in chlorophyll content, the reduction rate is related to the resistance of plants to salinity. Therefore, the chlorophyll content is a biochemical trait to show salt tolerance of different plants [38]. Under abiotic stresses, carotenoids play major role as a precursor in signaling pathways during the plant development because they protect cell membrane from oxidative stress [39].

**Fig. 3:**
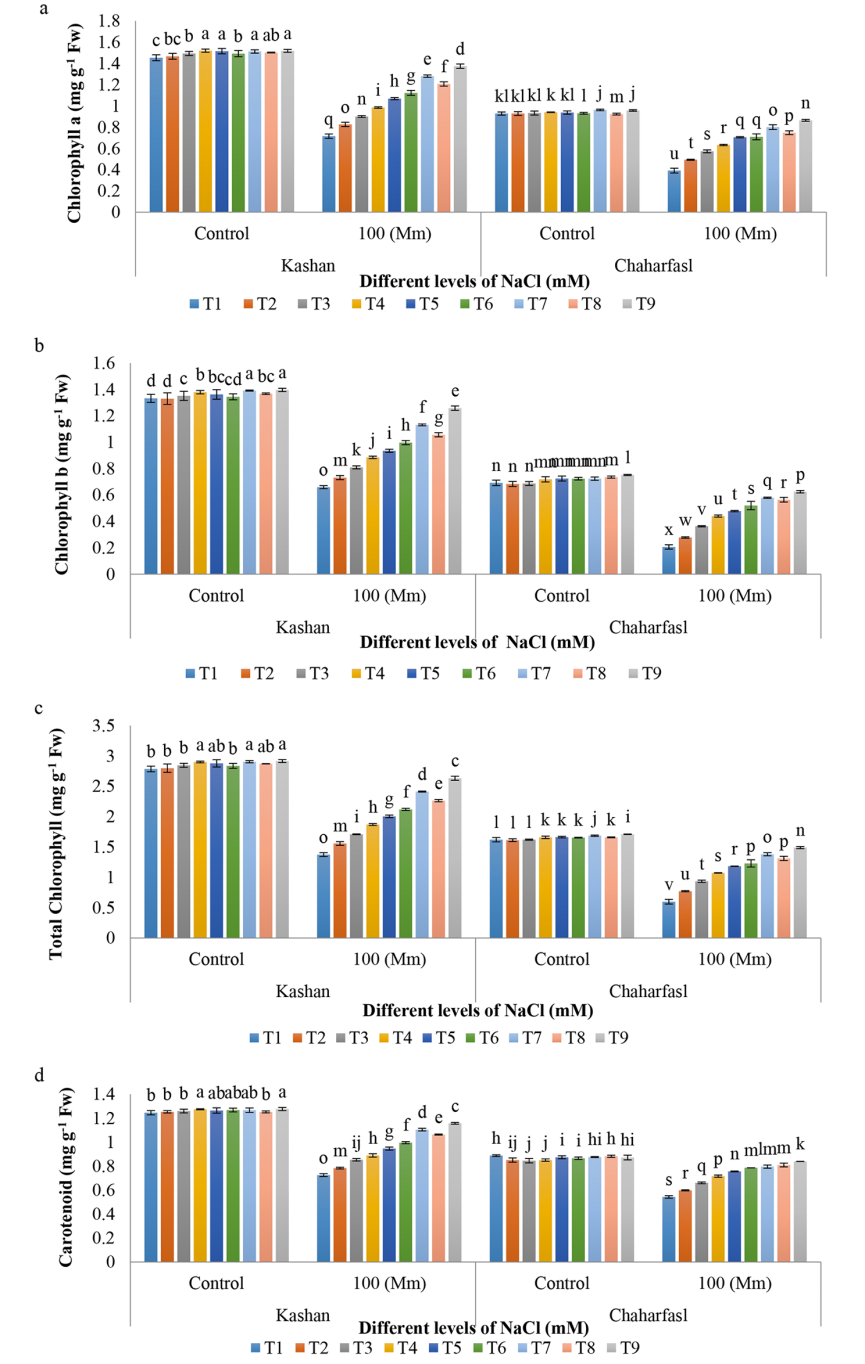
The interaction of in vitro induced-salt stress, nSiO_2_ and AnE on **a)** Chl *a*, **b)** Chl *b*, **c)** total Chl and **d)** carotenoid of *R. damascena* genotypes; **T1** (Control), **T2** (AnE 2 g/L), **T3** (AnE 3 g/L), **T4** (nSiO_2_ 50 mg/L), **T5** (nSiO_2_ 100 mg/L), **T6** (AnE 2 g/L + nSiO_2_ 50 mg/L), **T7** (AnE 2 g/L + nSiO_2_ 100 mg/L), **T8** (AnE 3 g/L + nSiO_2_ 50 mg/L), **T9** (AnE 3 g/L + nSiO_2_100 mg/L)

The results indicated that all treatments under control conditions had the highest Chl *a* compared to the salt treatments which were the lowest value in both genotypes, although the amount of Chl *a* in ʻKashanʼ was more than that in ʻChaharfaslʼ. The minimum value of Chl *a* (0.39 mg g^− 1^ Fw) was related to the control treatment in ʻChaharfaslʼ under 100 mM NaCl. However, the most suitable treatment in both genotypes under 100 mM NaCl was related to T9 treatment (AnE 3 g/L + nSiO_2_ 100 mg/L). On the other hand, under control conditions the effect of nSiO_2_ and its combination with AnE was more than AnE at 2 and 3 g/L concentrations (Fig. 3a). The maximum Chl *b* was observed in T7 (AnE 2 g/L + nSiO_2_ 100 mg/L) and T9 (AnE 3 g/L + nSiO_2_ 100 mg/L) treatments of ʻKashanʼ under control conditions, while control explants of ʻChaharfaslʼ had the least Chl *b* under 100 mM NaCl. In general, the application of salinity caused a significant decrease in Chl *b* compared to the control condition in both genotypes. The most suitable treatment in both genotypes under 100 mM NaCl was AnE (3 g/L) in combination with nSiO_2_ (100 mg/L). Also, ʻKashanʼ was more resistant to the 100 mM NaCl as the amount of Chl *b* reduction in ʻKashanʼ was lower than that in ʻChaharfaslʼ (Fig. 3b) (51% compared to 57% under control conditions). The same results were observed in total Chl as the total Chl value reduction in 100 mM NaCl was 102% and 174% in control explants of Kashan and Chaharfasl, respectively while treatment of the Damask explants with 3 g/L AnE + 100 mg/L nSiO_2_ reduced the mentioned reduction to 10% and 14% in the same genotypes (Fig. 3c). Salt stress caused a significant decrease in carotenoid levels in comparison with the control condition in both genotypes, although the amount of carotenoids was more in ʻKashanʼ compared to ʻChaharfaslʼ. The ameliorative effect of T9 treatment in both genotypes under 100 mM NaCl was more than other treatments as it decreased the carotenoid reduction in ‘Kashanʼ and ‘Chaharfaslʼ, respectively by 10% and 4% (Fig. 3d).

Chlorophyll holds a paramount role in plant pigmentation, primarily serving as the key pigment responsible for harnessing light energy in photosynthesis. A decrease in chlorophyll content can significantly curtail the rate of photosynthesis, thereby resulting in reduced crop yield [40]. Carotenoids have less sensitivity to stress than chlorophyll, which is probably due to the protection of the photosynthetic apparatus against stress, and carotenoids protect chlorophyll from photo-oxidation, as a result, the reduction of carotenoids can have serious effects on chlorophyll pigment [41]. Notably, silicon contributes to the preservation of chlorophyll within chloroplasts by maintaining the structural integrity of chloroplasts, particularly the grana. Furthermore, silicon enhances nutrient supply and the balance of macro and microelements within plant cells [42]. This translates to an increased availability of essential elements such as nitrogen, magnesium, and iron, all of which are integral to chlorophyll synthesis. Specifically, magnesium and nitrogen are integral components of chlorophyll’s molecular structure, while iron is essential for its formation. Silicon also appears to augment chlorophyll content by increasing the levels of α-aminolinolenate, a precursor of chlorophyll. Several studies have substantiated the role of *A. nodosum* extract in augmenting leaf chlorophyll content, attributable to the presence of aminobutyrate, glycine betaine, and betaine within the extract [43, 44].

**Fig. 4:**
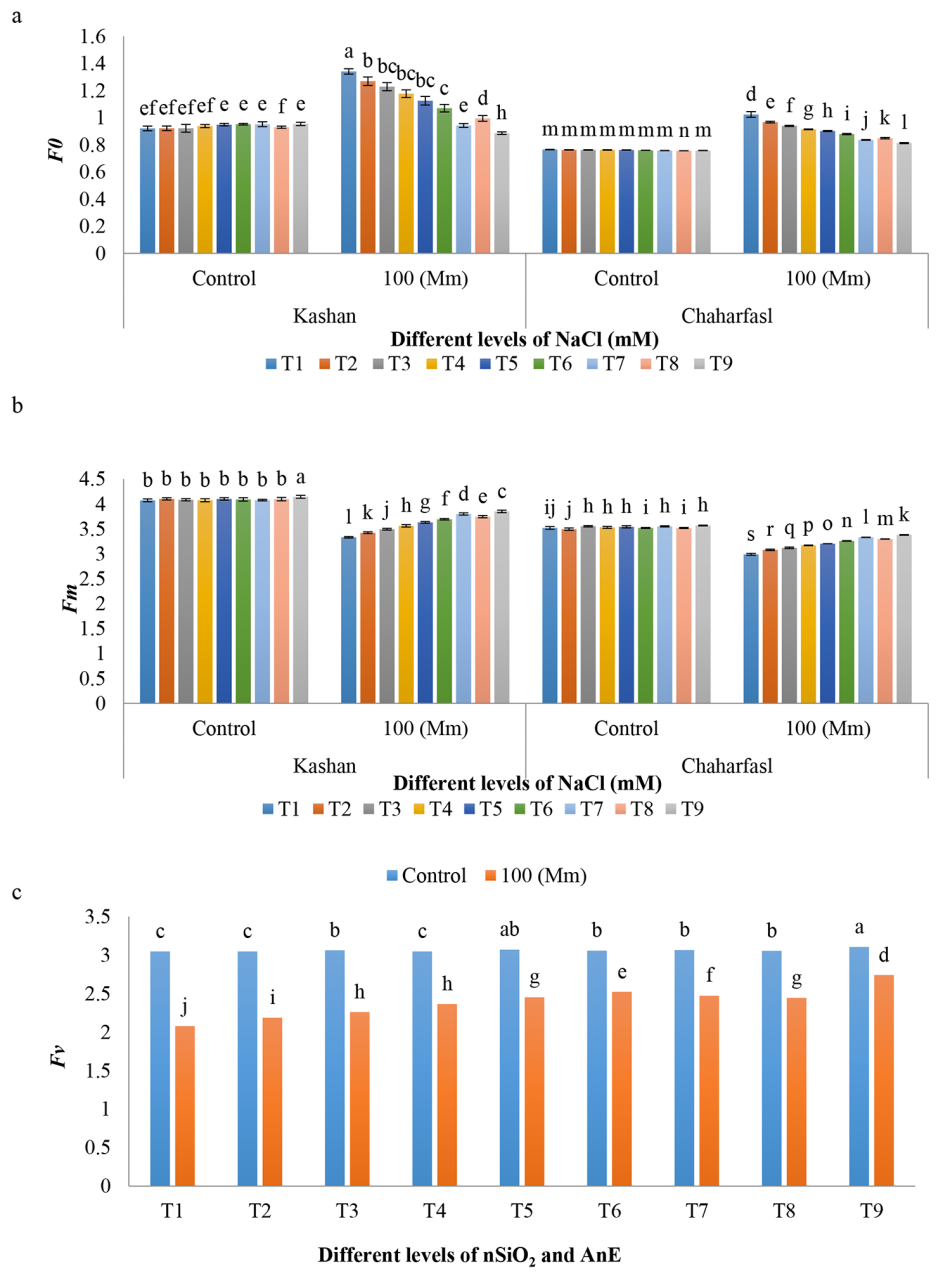
The interaction of in vitro induced-salt stress and SiO_2_-NPs and AnE on **a)** *F0*, **b)** *Fm*, and **c)** *Fv* of *R. damascena* genotypes; **T1** (Control), **T2** (AnE 2 g/L), **T3** (AnE 3 g/L), **T4** (nSiO_2_ 50 mg/L), **T5** (nSiO_2_ 100 mg/L), **T6** (AnE 2 g/L + nSiO_2_ 50 mg/L), **T7** (AnE 2 g/L + nSiO_2_ 100 mg/L), **T8** (AnE 3 g/L + nSiO_2_ 50 mg/L), **T9** (AnE 3 g/L + nSiO_2_100 mg/L)

**Fig. 5:**
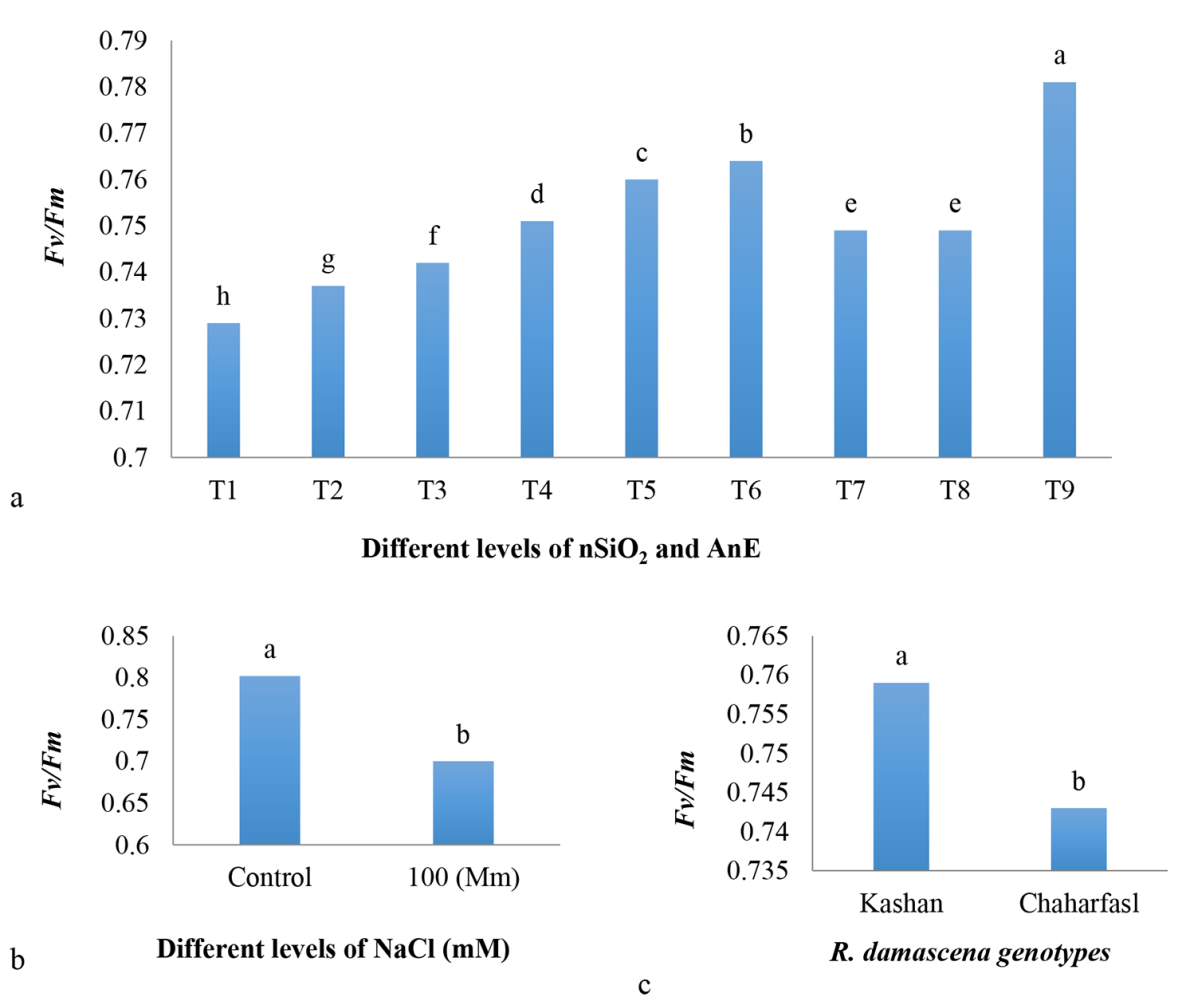
The effect of nSiO_2_, and AnE on **a)** *Fv/Fm*, **b)** The effect of in vitro induced salt stress on *Fv/Fm*, and **c)** The effect of *R. damascena* genotypes on *Fv/Fm*; **T1** (Control), **T2** (AnE 2 g/L), **T3** (AnE 3 g/L), **T4** (nSiO_2_ 50 mg/L), **T5** (nSiO_2_ 100 mg/L), **T6** (AnE 2 g/L + nSiO_2_ 50 mg/L), **T7** (AnE 2 g/L + nSiO_2_ 100 mg/L), **T8** (AnE 3 g/L + nSiO_2_ 50 mg/L), **T9** (AnE 3 g/L + nSiO_2_100 mg/L)

Salinity caused a significant increase in *F0* compared to the control condition in both genotypes (Fig. 4a). The maximum *Fm* value with a significant difference compared to other treatments was observed in T9 treatment (AnE 3 g/L + nSiO_2_ 100 mg/L) under control conditions in ‘Kashanʼ while T1 (control) treatment in ‘Chaharfaslʼ under 100 mM NaCl had the lowest *Fm*. Salinity caused a significant decrease in *Fm* compared to the control condition in ʻKashanʼ and ʻChaharfaslʼ by 22% and 17%, respectively. Application of AnE (3 g/L) + nSiO_2_ (100 mg/L) was the most suitable treatment in preventing of *Fm* reduction as it increased the *Fm* value by 15% and 13% in ʻKashanʼ and ʻChaharfaslʼ, respectively compared to the controls under saline conditions (Fig. 4b). It is suggested that ʻKashanʼ was more resistant to salinity than ʻChaharfaslʼ as it had the minimum fluorescence reduction than the other one (Fig. 4b). Salinity caused to decrease in the efficiency of the photosystem II (*Fv/Fm*) compared to the control conditions. ʻKashanʼ had the maximum *Fv/Fm* value compared to the other genotype. The ameliorative effect of AnE (3 g/L) + nSiO_2_ (100 mg/L) was more than control and also other treatments, which is followed by AnE (2 g/L) + nSiO_2_ (50 mg/L), while the control explants had the least *Fv/Fm* (Fig. 5a, b and c). Based on the Fig. 4c, results showed that application of 100 mg/L nSiO_2_ (T5) and AnE (3 g/L) + nSiO_2_ (100 mg/L) (T9) under control conditions had the maximum *Fv* compared to other treatments. On the other hand, under salinity, *Fv* decreased significantly in both genotypes in comparison with the control conditions up to the least amount in control explants (2.07). The highest (2.7) *Fv* was recorded in ʻKashanʼ under control conditions, whereas the lowest (2.2) *Fv* was observed in ʻChaharfaslʼ under 100 mM NaCl (Fig. 4c).

**Fig. 6:**
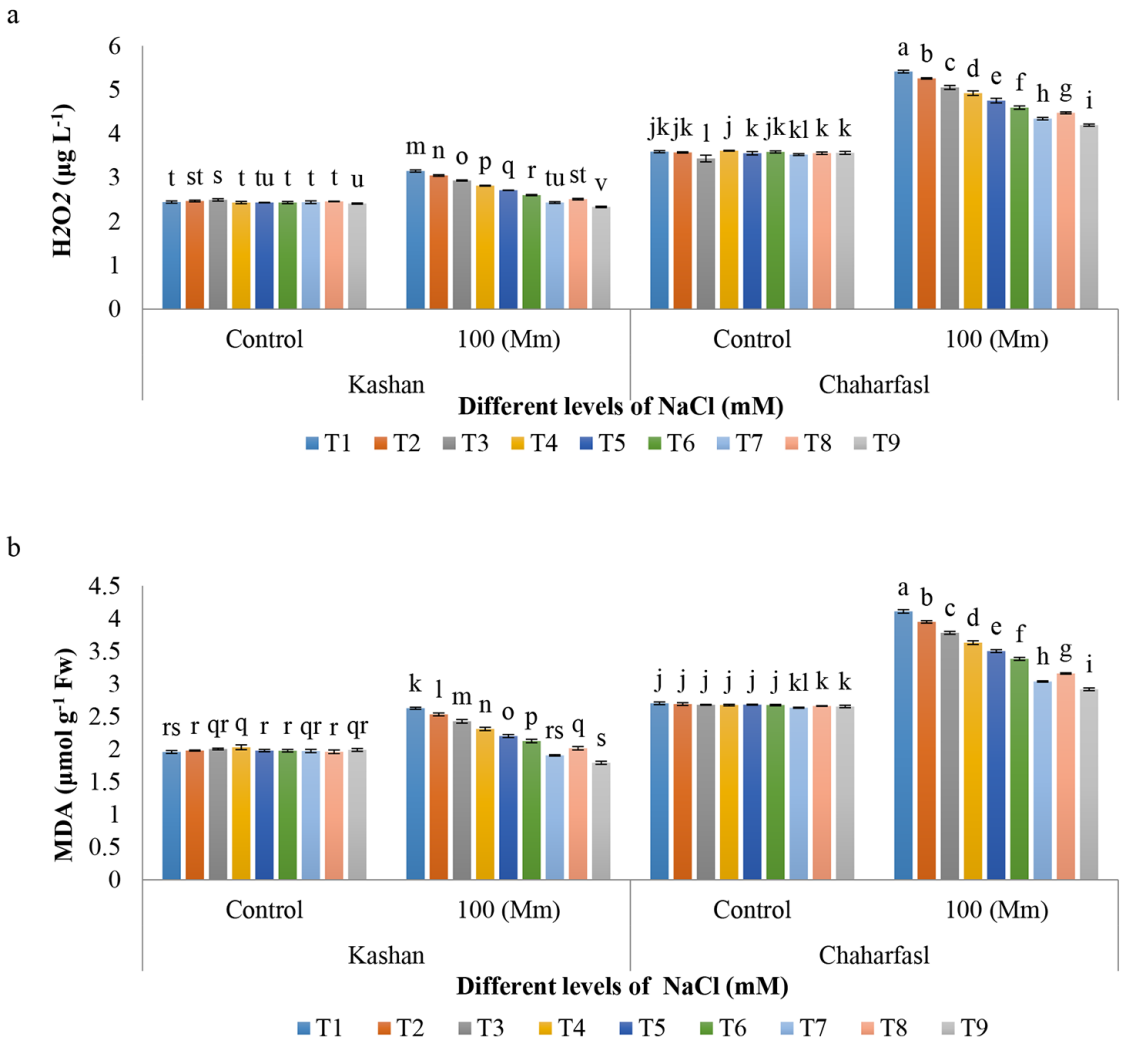
The interaction of in vitro induced-salt stress and nSiO_2_ and AnE on **a)** H_2_O_2_ and **b)** MAD of *R. damascena* genotypes; **T1** (Control), **T2** (AnE 2 g/L), **T3** (AnE 3 g/L), **T4** (nSiO_2_ 50 mg/L), **T5** (nSiO_2_ 100 mg/L), **T6** (AnE 2 g/L + nSiO_2_ 50 mg/L), **T7** (AnE 2 g/L + nSiO_2_ 100 mg/L), **T8** (AnE 3 g/L + nSiO_2_ 50 mg/L), **T9** (AnE 3 g/L + nSiO_2_100 mg/L)

The interaction effect of in vitro-induced salinity and treatments on MAD and H_2_O_2_ content of *R. damascena* genotypes was significant (p ≤ 0.01) as the maximum MDA and H_2_O_2_ was recorded in control explants of ʻChaharfaslʼ followed by ʻKashanʼ under 100 mM NaCl in comparison with the other treatments, while the lowest value was related to all treatments in ʻKashanʼ in control conditions. In general, salinity caused a significant reduce in MDA and H_2_O_2_ content compared to the control conditions in both genotypes. However, the application of nSiO_2_ and AnE under salinity caused to decrease in MDA and H_2_O_2_. The best treatment in preventing of MDA and H_2_O_2_ increment in both genotypes, was related to T9 (AnE 3 g/L + nSiO_2_ 100 mg/L) which showed the least MDA and H_2_O_2_ as illustrated in Fig. 6a and b. Also, ʻKashanʼ assumed to be more tolerant to the salinity than ʻChaharfaslʼ because of having the lowest MDA and H_2_O_2_ values. ROS in plant cells cause various mechanisms to be activated in the plant to decrease the toxic effects of oxidative stress caused by salt stress. The common features of oxidative damage resulting from salinity is cell damage and changes in membrane permeability and peroxidation of membrane lipids. This is accompanied by MDA production which can be considered as an indicator of the level of salinity damage. Fatty acids and lipids are very sensitive to ROS and oxidize quickly. Silicon reduces the peroxidation of cell membrane lipids and decreases MDA content by (1) scavenging ROS directly and (2) reducing the permeability of the cell membrane, and consequently increasing antioxidative enzymes activity, indirectly. The presence of H_2_O_2_ in the plant is important for alarm reactions in plant as in moderate concentrations, it acts as a signal molecule and participates in the production of cell wall protein precursors. However, the level of H_2_O_2_ in the plant increases across abiotic stresses which is toxic to the plant and causes oxidative damage. ROS potentially has the ability to react with many cellular compounds and cause damage to the membrane and other macro macromolecules including lipids, nucleic acids, proteins and photosynthetic pigments, [45]. Silicon reduces the H_2_O_2_ content and improves some of the unfavorable effects of various stresses in plant tissues by increasing the activity of antioxidative enzymes like catalase [46]. Furthermore, phytoalexin is one of the important compounds found in algae extract, which can be considered for its anticancer and antioxidant effects and its role in neutralizing free radicals [47]. Extensive literature supports the idea that sulfated polysaccharides in seaweed possess substantial antioxidant capacity. These compounds induce defense responses in plants. Laminarin, derived from seaweed, positively impacts the plant’s defense system by stimulating the production of essential growth regulators, antifungal compounds such as phytoalexins, and hydrolyzing enzymes like chitinase and gluconate. This protective effect underscores the crucial role of seaweed extracts in enhancing plant defenses [48].

**Fig. 7:**
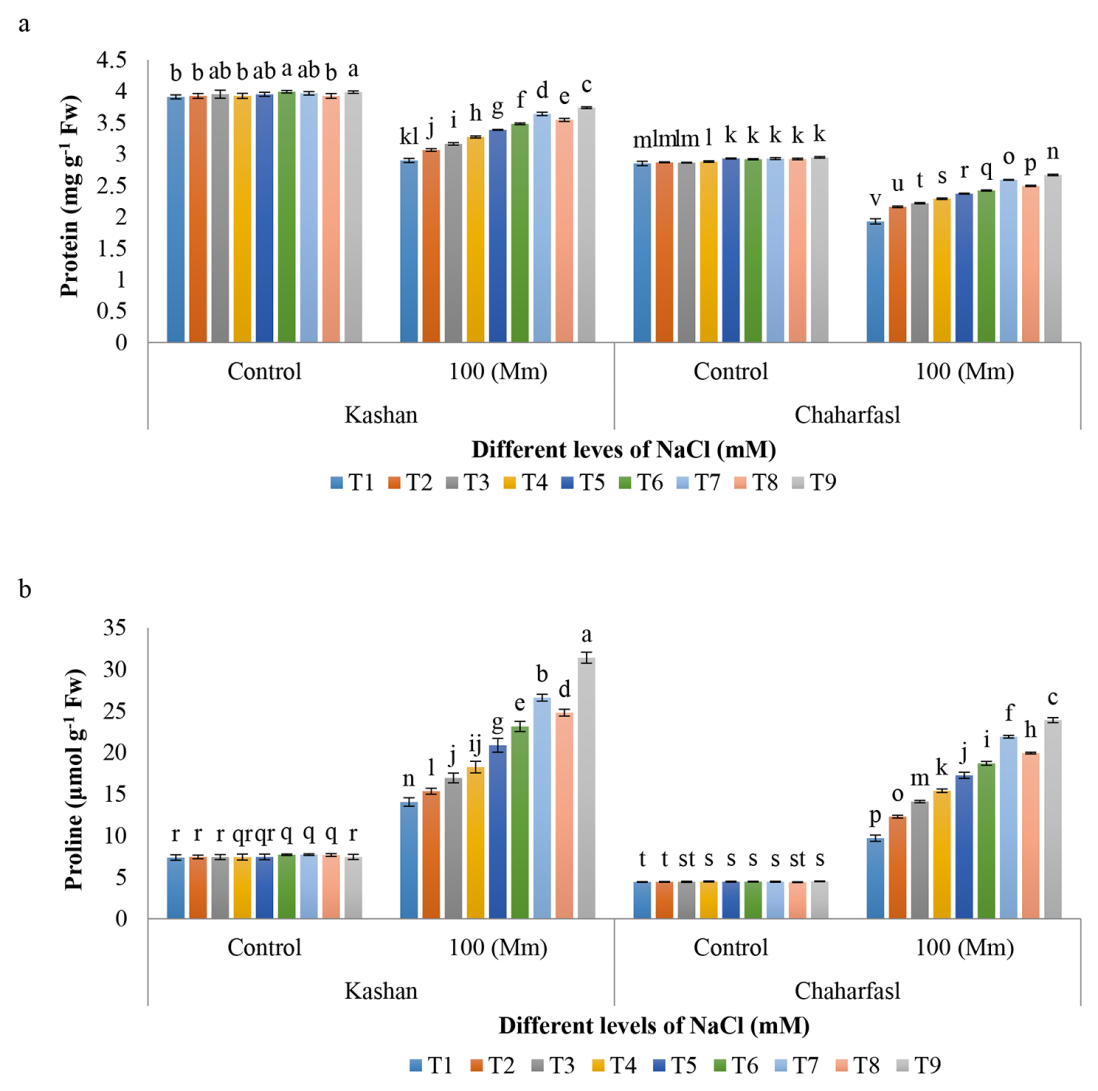
The interaction of in vitro induced-salt stress and nSiO_2_, and AnE on **a)** protein and **b)** proline content of *R. damascena* genotypes; **T1** (Control), **T2** (AnE 2 g/L), **T3** (AnE 3 g/L), **T4** (nSiO_2_ 50 mg/L), **T5** (nSiO_2_ 100 mg/L), **T6** (AnE 2 g/L + nSiO_2_ 50 mg/L), **T7** (AnE 2 g/L + nSiO_2_ 100 mg/L), **T8** (AnE 3 g/L + nSiO_2_ 50 mg/L), **T9** (AnE 3 g/L + nSiO_2_100 mg/L)

The interaction effect of in vitro-induced salinity and treatments on total protein and proline content of *R. damascena* genotypes was significantly different at p ≤ 0.05 and p ≤ 0.01 probability level, respectively. The maximum (3.9 mg g^− 1^Fw) protein was observed in ʻKashanʼ explants treated with 2 g/L AnE + 50 mg/L nSiO_2_ (T6) and 3 g/L AnE + 100 mg/L nSiO_2_ (T9) under control conditions, while control explants of ʻChaharfaslʼ had the least (1.9 mg g^− 1^Fw) protein content under salinity. Salt stress caused a significant decrease in total soluble protein content compared to the control conditions in both genotypes (Fig. 7a). Application of 3 g/L AnE + 100 mg/L nSiO_2_ effectively prevented of protein reduction in both *R. damascena* genotypes under salinity. ʻKashanʼ had the least reduction in total soluble protein compared to the ʻChaharfaslʼ under 100 mM NaCl. Proteins as well as the accumulation of free amino acids such as proline, are related to each other. In addition, carbonyl groups produced by protein oxidation increase under stress in plants [49]. Previous studies showed that there is a strong relationship between the concentration of silicon and the level of compounds associated with respiration and the number of amino acids. Researches indicate the important role of silicon as a signaling metabolite that is able to stimulate the re-distribution of amino acids [50]. Application of silicon plays a key role in modulating the use of 2-Oxoglutarate for amino acid metabolism. Therefore, it assumed that silicon increases protein storage [44]. Treatment of salt stressed – explants with silicon caused a decrease in the amount of carbonyl groups, which indicates a decrease in oxidative damage to the protein, and therefore the destructive effects of stress are reduced and the amount of protein is maintained by increasing the tolerance of the plant. In the present research, the use of silicon reduced the effect of salinity and decreased the rate reduced trend of protein content under salinity. In addition, AnE increased the amount of protein, which is probably because of the increase in the synthesis of new proteins caused by the occurrence of stress resistance genes or the decrease in the activity of protein-degrading enzymes [51].

Based on the Fig. 7b the maximum proline content was observed in ʻKashanʼ explants treated with T9 (AnE 3 g/L + nSiO_2_100 mg/L), followed by T7 (AnE 2 g/L + nSiO_2_ 100 mg/L) under 100 mM NaCl while the lowest proline content was related to all treatments of ʻChaharfaslʼ. In general, salinity caused a significant increase in proline content in both genotypes compared to the control conditions. Biosynthesis and accumulation of organic substances with low molecular weight, including proline is one of the tolerance mechanisms of plants against salt stress [52]. Proline serves as an osmotic protector, safeguarding enzymes, and cellular structures from osmotic stress. As sodium (Na^+^) and chloride (Cl^−^) concentrations rise in the rhizosphere, and consequently their absorption increases, plants transport these ions to vacuoles for neutralizing their toxic effects and establishing osmotic balance. This is achieved by accumulating organic compounds, like proline, in the cytoplasm [53]. Moreover, proline plays a role in scavenging hydroxyl radicals, maintaining membrane stability, preserving protein structure, and providing nitrogen and carbon under stress conditions. Silicon application stimulates proline synthesis, facilitating various processes such as enzyme protection, regulation of the cell’s redox potential, membrane phospholipid stabilization, protein preservation, and pH regulation under stress conditions. Moreover, the reduction in proline breakdown contributes to increased plant tolerance to stress [54].

**Fig. 8:**
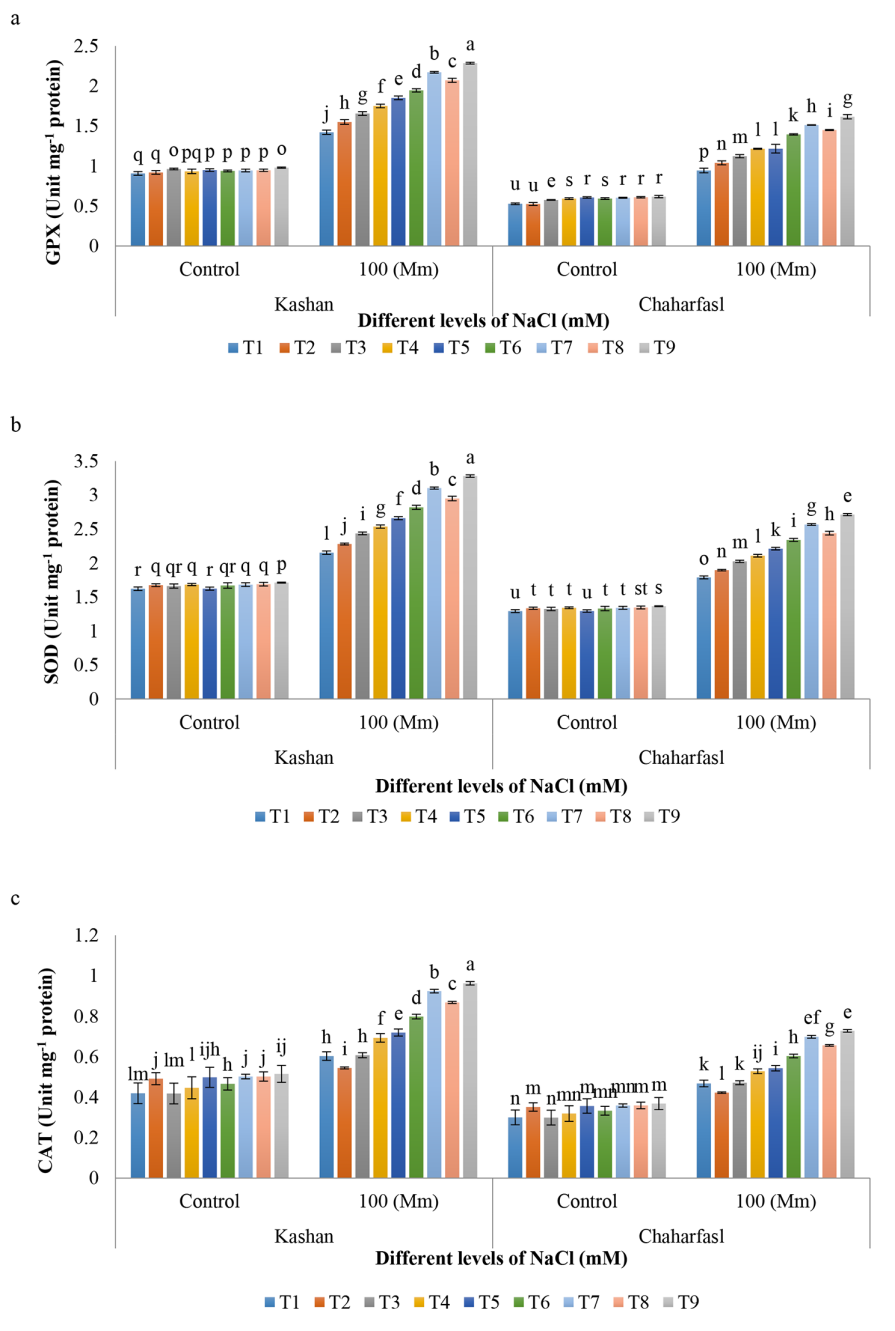
The interaction of in vitro induced-salt stress and nSiO_2_, and AnE on **a)** GPX, **b)** SOD, and **c)** CAT activity of *R. damascena* genotypes; **T1** (Control), **T2** (AnE 2 g/L), **T3** (AnE 3 g/L), **T4** (nSiO_2_ 50 mg/L), **T5** (nSiO_2_ 100 mg/L), **T6** (AnE 2 g/L + nSiO_2_ 50 mg/L), **T7** (AnE 2 g/L + nSiO_2_ 100 mg/L), **T8** (AnE 3 g/L + nSiO_2_ 50 mg/L), **T9** (AnE 3 g/L + nSiO_2_100 mg/L)

The triple interaction between salinity, genotype, and different treatments (nSiO_2_ and AnE) had a significant effect on GPX, SOD, and CAT. Salinity caused to a significant increase in GPX activity in both genotypes compared to the control condition. ʻKashanʼ explants treated with 3 g/L AnE + 100 mg/L nSiO_2_ showed the maximum GPX activity compared to other treatments under salt stress. The explants treated with 2 g/L AnE + 100 mg/L nSiO_2_ had also higher GPX activity compared to other treatments. However, the least GPX activity was related to all treatments of Chaharfasl genotype under control conditions (Fig. 8a). The same results were observed in the activity of SOD and CAT (Fig. 8b and c). SOD is one of the most important and effective intracellular antioxidants whereas many researchers demonstrated that SOD is the strongest known antioxidant that protects many plants against the attack of oxygen free radicals and oxidative stress damage [55]. It is also the main inhibitor of ROS, whose enzymatic activity causes the formation of O_2_ and H_2_O_2_ [56]. CAT caused to produce water and oxygen from H_2_O_2_, which is mainly found in cytosol, mitochondria and peroxisomes and prevents lipid oxidation, chlorophyll destruction and cell wall damage [57]. In general, using of different AnE and nSiO_2_ treatments and their combinations increased GPX, SOD and CAT activities more than control treatments under salt stress while under control conditions their effect were not so significantly different especially in ‘Chaharfaslʼ. However, AnE and nSiO_2_ treatments also increase the mentioned enzymes activity under control conditions (no salt stress).

**Fig. 9:**
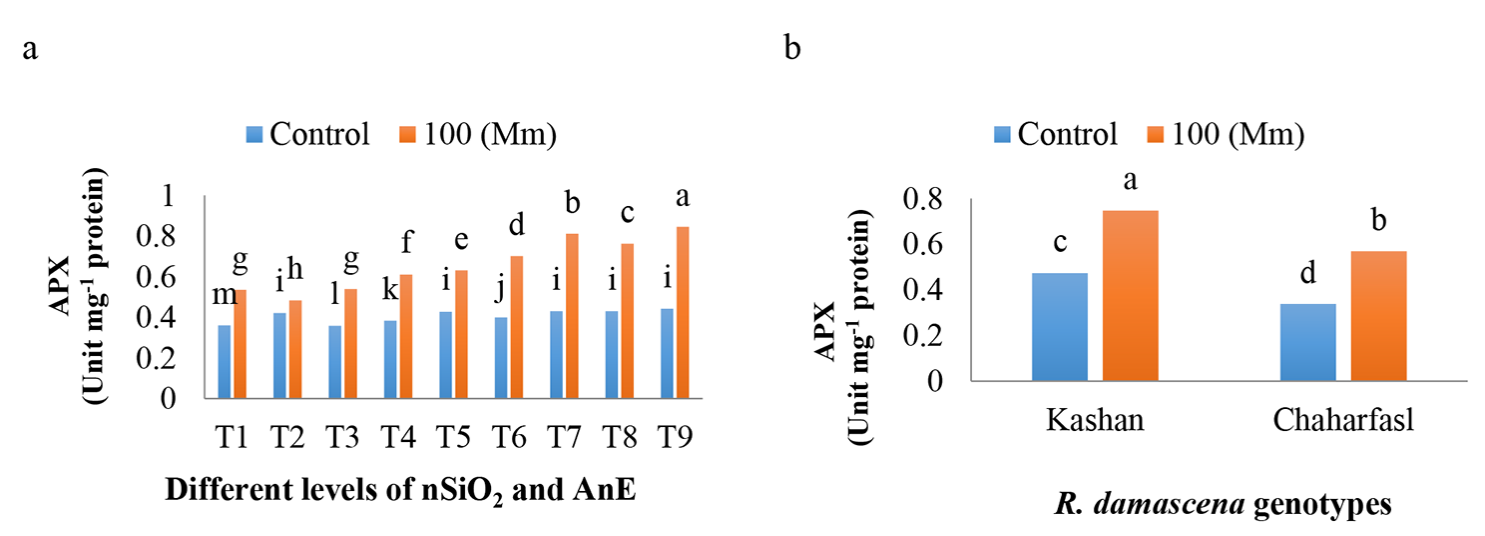
**a)** The interaction of in vitro induced-salt stress and nSiO_2_, and AnE extract and **b)** The effect of in vitro induced-salt stress on APX activity of *R. damascena* genotypes; **T1** (Control), **T2** (AnE 2 g/L), **T3** (AnE 3 g/L), **T4** (nSiO_2_ 50 mg/L), **T5** (nSiO_2_ 100 mg/L), **T6** (AnE 2 g/L + nSiO_2_ 50 mg/L), **T7** (AnE 2 g/L + nSiO_2_ 100 mg/L), **T8** (AnE 3 g/L + nSiO_2_ 50 mg/L), **T9** (AnE 3 g/L + nSiO_2_100 mg/L)

The effect of in vitro induced-salt stress on APX activity was illustrated in Fig. 9a and b. APX activity increased under 100 mM NaCl and interestingly the different treatments also increased its activity so that *R. damascena* explants treated with 3 g/L AnE + 100 mg/L nSiO_2_ showed maximum APX activity (Fig. 9a). Comparing the APX activity in two genotypes revealed the increased APX activity in ‘Kashanʼ and ‘Chaharfaslʼ by about 1.5 folded (Fig. 9b).

It is demonstrated that seaweed extract causes to increase in shoot and root length, root and shoot area, and shoot and root fresh weight either in control conditions or salt stress conditions because of having signaling molecules and various bioactive compounds, along with organic and mineral nutrients, that greatly is beneficial for the plants [58]. On the other hand, it has potential to enhance the plant root morphology and also productivity by making the plants ability to adequately uptake nutrients from the deeper soil layers especially under salinity [59].

The heightened activity of APX during salt-induced stress, as supported by previous findings from Hernández-Herrera et al. [58], underscores the plant’s response to oxidative stress. While the application of AnE did not lead to a substantial increase in the activity of APX beyond that observed under salt stress conditions, it is worth noting that AnE contains a rich composition of minerals and antioxidants, including folic acid. These components play a pivotal role in mitigating the toxic effects of ROS and enhancing overall plant productivity by facilitating increased nutrient uptake and availability [60].

### Pearson correlation analysis of *R. damascena* genotypes to the nSiO_2_ and AnE treatments unde r in vitro induced-salt stress

**Fig. 10:**
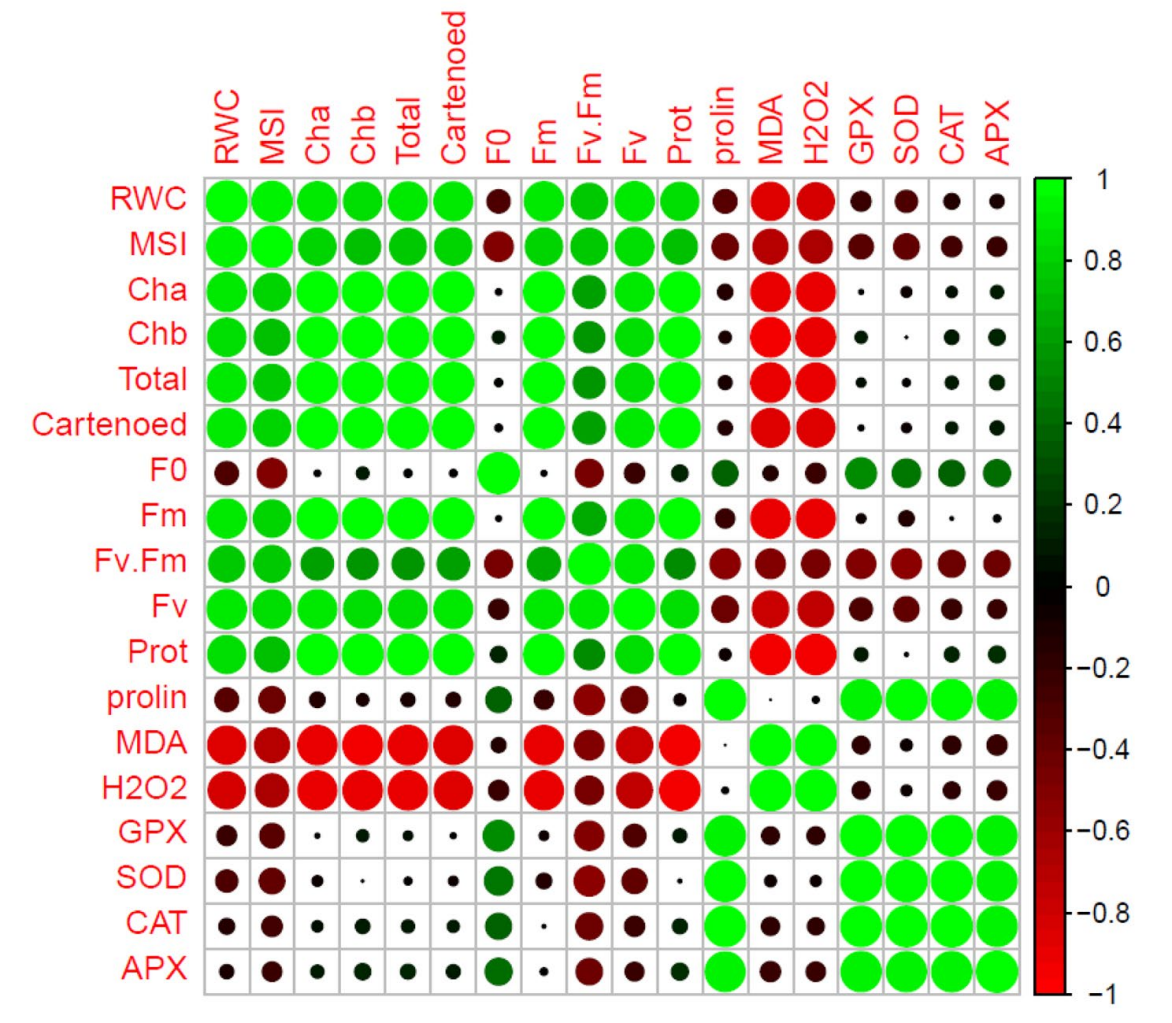
PCA of nSiO_2_ and AnE treatments and variable trait relationship in *R. damascena* under control and 100 mM NaCl. The heat map of the PCA coefficient (r) values of variable traits, where the colored scale indicates negative (red) or the positive (green) correlation and the ‘r’ coefficient values (r = − 1.0 to 1.0). The tested variables showed Chl *a*, maximal fluorescence from dark-adapted leaf (*Fm*), carotenoids (CARs), maximum PSII (*Fv/Fm*), Total Chl, Chl *b*, protein (Pro), membrane stability index (MSI), relative water content (RWC), superoxide dismutase (SOD), variable fluorescence (*Fv*), guaiacol peroxidase (GPX), proline (Pro), malondialdehyde (MDA), initial fluorescence (*F0*), hydrogen peroxidase (H_2_O_2_), ascorbate peroxidase (APX), and catalase (CAT)

Firstly, there were positive correlations observed between photosynthetic pigments and several key parameters, including MSI, RWC, protein content, and chlorophyll fluorescence parameters. These positive correlations suggest that photosynthetic pigments, crucial for plant photosynthesis, were linked to the overall health and functional attributes of the plant, reflecting their positive response to the applied treatments. Conversely, H_2_O_2_, proline, and MDA exhibited positive correlations among themselves. These components, typically associated with stress responses and oxidative damage, represented a negative correlation with protein content, photosynthetic pigments, and chlorophyll fluorescence parameters. This suggests that an increase in oxidative stress indicators coincided with a decrease in the above-mentioned parameters, signifying the detrimental impact of salt stress on these aspects of plant physiology. Furthermore, the antioxidant content within the samples, including CAT, GPX, APX, and SOD, exhibited significant positive correlations with one another. This implies a coordinated response of these antioxidants in modulating the effects of salt-induced oxidative stress. Certain traits, such as the total soluble protein content, RWC, individual chlorophyll components (Chl *a* and *b*), Carotenoids (CARs), total chlorophyll (total Chl), and various chlorophyll fluorescence parameters (*Fm*, *Fv/Fm*, and *Fv*), displayed negative correlations with salinity-induced stress. However, the application of nSiO_2_ and AnE treatments appeared to counteract the negative effects of salt stress, as evidenced by the modulation of these traits. These findings suggest that nSiO_2_ and AnE treatments have the potential to ameliorate the adverse effects of salinity on these crucial plant attributes [Fig. 10].

## Conclusion

Considering the changing weather patterns associated with global warming, there has been a notable increase in drought conditions, leading to elevated soil salinity levels. This environmental challenge, particularly relevant in the context of rose cultivation, necessitates an understanding of salt stress tolerance thresholds and defense mechanisms, as it has a profound impact on plant health and survival. The findings derived from this study underscore the effective functioning of stress tolerance mechanisms within the tested *R. damascena* genotypes, even in the face of severe salt stress. These mechanisms encompass osmotic and antioxidant protection processes, which play pivotal roles in safeguarding the explants from damage and deterioration. Of particular significance is the observation that the ‘Kashan’ displayed superior tolerance to salt stress. This enhanced tolerance was attributed to its remarkable antioxidant activity, the accumulation of compatible solutes, and the notably high concentration of total soluble protein relative to the other genotype. This distinctive performance may be inherent to the genetic makeup of the ‘Kashan’. In summary, the results highlight the efficacy of in vitro selection methods for screening salt-tolerant *R. damascena* genotypes. Nevertheless, to validate the in vitro experimental findings, it is advisable to conduct parallel experiments in open field conditions. The outcomes of this investigation emphasize the pivotal role of antioxidant defense systems, such as flavonoids, total phenols, and proline, as well as optimized photosynthetic performance, in modulating the responses to salinity tolerance, particularly in the context of treatments involving nSiO_2_ and AnE. These treatments, which hold promise as cost-effective and environmentally friendly alternatives to traditional fertilizers, demonstrate their potential in enhancing salt stress resilience. The combination of nSiO_2_ (100 mg/L) and AnE (at concentrations of 2 g/L or 3 g/L) emerges as the most effective treatment in modulating the deleterious effects of salt stress. Additionally, the study indicates that AnE alone can yield positive outcomes, underscoring the synergistic effects of nSiO_2_ and AnE in enhancing salinity tolerance in *R. damascena*.

## Materials and methods

### Plant materials and culture medium

Cuttings of two distinct genotypes of *Rosa damascena*, ʻChahar-fasalʼ and ʻKashanʼ, were sourced from a greenhouse located in Mahallat, Iran, at coordinates 33.9115° N and 50.4525° E. These cuttings were initially cultivated within a greenhouse environment. Following an incubation period of two months, the genotypes of *R. damascena*, represented by one-year-old branches exhibiting active growth, were carefully excised. Subsequently, these branches underwent a thorough 20-min washing with tap water and were then subjected to a 20-min surface sterilization process utilizing a 30% (v/v) NaClO solution. The branches were subsequently trimmed to yield one-node shoot explants, measuring between 1.5 and 2 cm in length. These explants were placed into a hormone-free MS (Murashige and Skoog) medium for their initial establishment, which was solidified using 7.5 g of agar [61]. The detailed composition of each medium element can be found in supplementary Table 1. Prior to autoclaving at 121 °C for 20 min, the pH value of the medium was adjusted to 5.7. Following the pH adjustment, the samples underwent autoclaving at 121 °C for a duration of 20 min. Subsequently, five shoot explants were cultured within 150 mL vessels having 25 ml of the MS medium in each of them. These cultures were maintained under controlled conditions, characterized by a temperature of 25 ± 2 °C, a photoperiod of 16 h (with a light intensity of 8.85 W/m²), and relative humidity levels ranging from 60 to 70%. Daily surveillance of the explants was conducted to promptly identify any instances of fungal or bacterial contamination, leading to the removal of contaminated vessels from the experimental collections. The plant shoots were subjected to three successive subcultures, with a consistent subculture interval of three weeks between each cycle.

***Proliferation medium.*** The foundational medium employed for the proliferation media was consistent with the MS establishment medium [61]. This medium consisted of 332 mg of calcium chloride (CaCl_2_), supplemented with an additional 130 mg of iron sequester, in addition to 0.03 mg L^− 1^ of Indole 3-butyric acid (IBA) and 0.36 mg L^− 1^ of benzyl adenine (BA). The pH of the medium was adjusted to 5.7 prior to undergoing sterilization in an autoclave. For subculturing purposes, 25 mL of this medium was dispensed into the vessels. Subsequently, the vessels, with the medium, were subjected to sterilization and then transferred to a controlled environment within a laminar flow hood. Following these preparations, five shoot nodes were individually placed in 150 mL culture vessels having the proliferation medium as mentioned above. These vessels constituted a single replication and were subsequently transferred to a growth chamber for the propagation process.

***Implementation of NaCl-induced salinity and treatments***: To induce salt stress in the culture medium, sodium chloride (NaCl) was introduced at two concentrations: 0 mM and 100 mM, in accordance with prior research [10], to replicate salinity conditions. Subsequently, the treatment involved the addition of nano-silicon (nSiO_2_) at three different levels: 100 mg L^− 1^, 50 mg L^− 1^, and a null (0) concentration. Simultaneously, *Ascophyllum nodosum* extract (AnE) was introduced at three varying levels: 0 g L^− 1^, 2 g L^− 1^, and 3 g L^− 1^. The nSiO_2_, characterized by particles smaller than 50 nm, were sourced from Nanosany Corporation, a nanomaterial company based in Iran as described in our last work [62], while the AnE was procured from Hamyar Dasht ABROON in Iran. Further details regarding the properties of *Ascophyllum nodosum* seaweed extract can be found in supplementary Table 2. Following the placement of shoot explants, each possessing four young leaves, into the culture medium, five shoot nodes were placed in each culture dish as illustrated in Fig. 1 of supplementary file. These dishes were subsequently exposed to a controlled growth chamber and kept at 25 ± 2 °C, under a 16-h photoperiod with a light intensity of 8.85 W/m^2^, and relative humidity ranging from 60 to 70%. After a period of 30 days, during the harvest, various physiological and biochemical properties were assessed for the collected explants.

### Physiological traits

#### Relative water content (RWC)

The explant leaves were weighed as fresh weight (Fw) and then submerged in ddH_2_O at 4 °C for 24 h. The samples were re-weighed for recording turgid weight (TW) immediately after removing the surface water. Then, after drying the samples in the oven at 75 °C for 48 h, the DW was recorded and the RWC was measured according to the method of Barrs and Weatherley [63].

#### Membrane stability index (MSI)

For measuring the stability of membrane, 0.1 g of *R. damascena* leaves were weighed. Two sets of the same samples were detached and put in test tubes containing 10 mL of dH_2_O. One set of the samples were incubated at 40 °C for 30 min (C1) and the other set were kept at 100 °C for 15 min (C2) and the EC were read for each of the via EC meters (Jenway model, UK) [64].

#### Total chlorophyll, chlorophyll b, a, and carotenoids (CARs)

The extract of 0.5 g of leaf samples were obtained by use of 10 mL of 80% acetone until the leaves completely bleached and chlorophyll was detached after 24 h of incubation in a dark place. Thus, the absorbance amount of the extracted samples was determined by spectrophotometer (Shimadzu UV-2550, Japan) according to the Arnon [65] protocol.

#### Chlorophyll fluorescence parameters

The leaf chlorophyll fluorescence of the samples was recorded by photosynthesis meter (Walz GmbH Licensing, Germany). *F0* (minimum fluorescence), was measured in the leaves after 30 min’ incubation in a dark room and then used with the leaves in the full light position to calculate the maximum fluorescence *Fm*. Photochemical performance (*Fv/Fm*) and maximal variable fluorescence (*Fv*) of PSII were calculated according to the detected parameters [66].

## Biochemical traits

### Antioxidative enzyme activities

To determine the antioxidative enzyme activities, fresh leaves (1 g) were homogenized and crushed by 50 mM (pH 7.0) K–phosphate buffer containing 1 M sodium chloride, 1% polyvinyl pyrrolidine and 1 mM EDTA. Then the mixed extract was centrifuged at 20,000 g (Z 216 MK model, Germany) for 15 min at 4 °C and the supernatants were taken to determine the enzyme activities in samples by means of spectrophotometer (UV-1800 Shimadzu, Japan). Guaiacol peroxidase (GPX) content of samples during polymerization of guaiacol was recorded by calculating the amount of extract absorbance at 470 nm (ε = 26.6 mM^− 1^ cm ^− 1^). Superoxide dismutase (SOD) content of samples was calculated by the method previously described by Beauchamp and Fridovich [67]. Then, samples in reactant tubes kept under a 20 W fluorescent lamp for 15 min were covered with a black cloth and the amount of absorbance was read at 560 nm. For measuring the activity of catalase (CAT) in the samples, the protocol of Li et al. [68] was used. In this case, 0.5 g of explant leaves were rounded in liquid nitrogen and mixed with 100 mM potassium phosphate buffer (pH 7.8) and the extracted solution was centrifuged at 10,000 rpm for 15 min at 4 °C. Then the supernatant was taken for measuring the enzyme activity. The activity of ascorbate peroxidase (APX) was measured in the solution extracted with phosphate buffer (250 µL), 1 mM ascorbate (250 µL), 0.4 µM EDTA (250 µL), 190 µL ddH_2_O, 10 mM trans oxide (10 µL), and 50 µL supernatant. Then, the absorbance value of the extract was read at 290 nm for 1 min.

### Protein content

For measuring the amount of protein in the explants, the protocol described by Bradford [69] was applied. For this reason, 100 mg of sample explants were mixed with 2 mL potassium phosphate buffer (50 mM) at pH 7.0 and then centrifuged at 7000–12,000 rpm. The supernatants were then collected and the amount of absorbance was recorded at 595 nm.

### Proline

For assaying the amount of proline, 0.2 g of leaf samples were homogenized in 2 mL aqueous sulfosalicylic acid (3%). Samples were centrifuged at 10,000 rpm for 30 min and after twice washing the pellet with aqueous sulfosalicylic acid, supernatant was pooled and measured by toluene extraction and ninhydrin reagent [70].

### Hydrogen peroxide (H_2_O_2_)

the amount of explants H_2_O_2_ was evaluated by means of Liu et al. [71] method. 0.5 g of explant leaves were grounded with potassium phosphate buffer (pH 6.8) and the leave the solution to rest for 30 min. Then 100-µL of the supernatants were mixed with 1 mL of Xylenol and the solution absorbance was recorded at 560 nm.

### Malondialdehyde (MDA)

For assaying the amount of MDA, 1.5 mL of the leaf extracts were added to 2.5 mL TBA (5%) and homogenized completely [72]. The solution was kept at 95 °C for 15 min and then cooled quickly and centrifuged at 5000 rpm for 10 min. Then the amount of supernatant absorption was recorded at 532 nm.

### Statistical analysis

The factorial experiment was done according to a completely randomized design with five explants in each plot and 3 replications and. Data for parameters were statistically analyzed by MSTAT-C ver 2.1 software and means were compared using the Duncan the levels of five and one% error probability. Pearson correlation and cluster dendrogram heat maps were performed in R foundation for statistical computing (version 4.1.2).

### Electronic supplementary material

Below is the link to the electronic supplementary material.

Supplementary Material 1

## Data Availability

Correspondence and requests for materials should be addressed to H.S.H.
