# Supplementary material for: Nano-silicone and Ascophyllum nodosum-based biostimulant down-regulates the negative effect of in vitro induced-salinity in Rosa damascena
Source: BMC Plant Biol. 2023 Nov 14;23:560. doi: 10.1186/s12870-023-04584-2 (PMC10644502; doi:10.1186/s12870-023-04584-2)

**Supplementary Table 1.** Presents the composition of the culture medium utilized in the experiments, with concentrations expressed in milligrams per liter (mg L^-1^). The culture medium is divided into several categories based on the types of compounds included.

| Concentration | **Compounds** |
| --- | --- |
| MS (mg L^-1^) | **Macro elements** |
| 1900 | KNO_3_ |
| 1650 | NH_4_NO_3_ |
| 440 | CaCl_2_.2H_2_O |
| 370 | MgSO_4_.7H_2_O |
| 170 | KH_2_PO_4_ |
| MS (mg L^-1^) | **Microelements** |
| 16.9 | MnSO_4_.7H_2_O |
| 8.63 | ZnSO_4_.7H_2_O |
| 6.2 | H_3_BO_3_ |
| 0.83 | KI |
| 0.25 | Na_2_MoO_4_.2H_2_O |
| 0.025 | CuSO_4_.5H_2_O |
| 0.025 | CoCl_2_.6H_2_O |
| MS (mg L^-1^) | **Fe** |
| 37.3 | Na_2_EDTA |
| 27.8 | FeSO_4_.7H_2_O |
| MS (mg L^-1^) | **Vitamins** |
| 100 | Mioinositol |
| 2 | Glycine |
| 0.5 | Thiamine |
| 0.5 | Pyridoxine |
| 0.5 | Niacine |

| **Supplementary Table 2.** Some properties of seaweed extract *Ascophyllum nodosum* (https://gb.kompass.com/c/hamyar-dasht-abroon/ir092333/) | | | | | | | | | | |
| --- | --- | --- | --- | --- | --- | --- | --- | --- | --- | --- |
| **EC**  **(dS m^-1^)** | **pH** | **Folic acid**  **(%)** | | **P**  **(%)** | **Ca**  **(%)** | **K**  **(%)** | **Fe**  **(%)** | **Total N**  **(%)** | **Relative density (g/cm^3^)** | **Organic matter (%)** |
| 1.3 | 9.2 | | 10.5 | 1 | 0.18 | 16.9 | 0.5 | 2.5 | 1.28 | 15 |

**Supplementary Figure 1.** The Damask proliferated shoots after a three-week period show that there is significant growth.

**
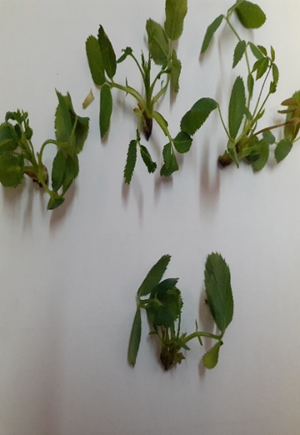
**  **
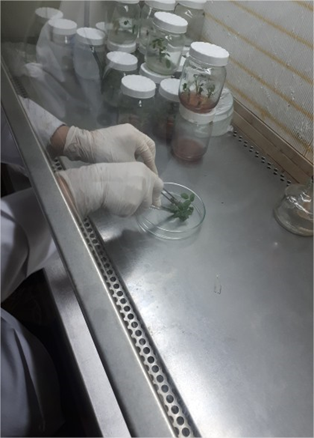
**


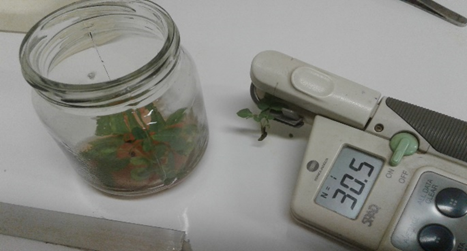

Supplement: Supplementary file 1 — Supplementary Material 1 [file 12870_2023_4584_MOESM1_ESM.docx]
